# Genetic, Immunological, Dietary, Gut Microbiota, and Environmental Determinants of Osteoporosis in the Course of Celiac Disease: Which Factor Plays the First Violin in This Orchestra?

**DOI:** 10.1007/s00223-023-01155-3

**Published:** 2023-12-05

**Authors:** Kinga Skoracka, Szymon Hryhorowicz, Francesco Tovoli, Alberto Raiteri, Anna Maria Rychter, Ryszard Słomski, Agnieszka Dobrowolska, Alessandro Granito, Iwona Krela-Kaźmierczak

**Affiliations:** 1 Department of Gastroenterology, Dietetics and Internal Diseases, Poznan University of Medical Sciences, 49 Przybyszewski Street, 60-355 Poznan, Poland; 2 Doctoral School, Poznan University of Medical Sciences, Fredry St. 10, 61-701 Poznan, Poland; 3 Institute of Human Genetics, Polish Academy of Sciences, Strzeszynska 32, 60-479 Poznan, Poland; 4 Division of Internal Medicine, Hepatobiliary and Immunoallergic Diseases, IRCCS Azienda Ospedaliero-Universitaria di Bologna, Bologna, Italy

**Keywords:** Celiac disease, Autoimmune disease, Immune-mediated disease, HLA, Osteoporosis

## Abstract

Celiac disease (CD) is a chronic small intestinal immune-mediated enteropathy precipitated by exposure to dietary gluten in genetically predisposed individuals. The worldwide prevalence of CD is estimated to be 0.7–1.4% of the general population. Etiopathology of this disease is multifactorial, with genetic determinants being a major contributing player to CD susceptibility. Its manifestation embraces different organs, including the musculoskeletal apparat. Patients with CD have increased risk of bone disorders. According to data, bone disorders – osteopenia and osteoporosis – can affect up to 70% of patients with CD at diagnosis, and it decreases after the initiation of a gluten-free diet. Gluten consumption in patients with CD triggers an inflammatory reaction followed by tissue damage, and both; local and systemic inflammation can increase the risk of bone mass deterioration. Other theory assumes shortages of vitamin D and an impaired calcium absorption mechanism leading to secondary hyperparathyroidism. Taking into account the increasing prevalence of CD and osteoporosis, we broadly discuss genetic, immunological, dietary, gut microbiota, and environmental factors that could increase the risk of osteoporosis in CD. Furthermore, we discuss lifestyle and pharmacological preventing and treatment measures.

## Introduction

**Fig. 1:**
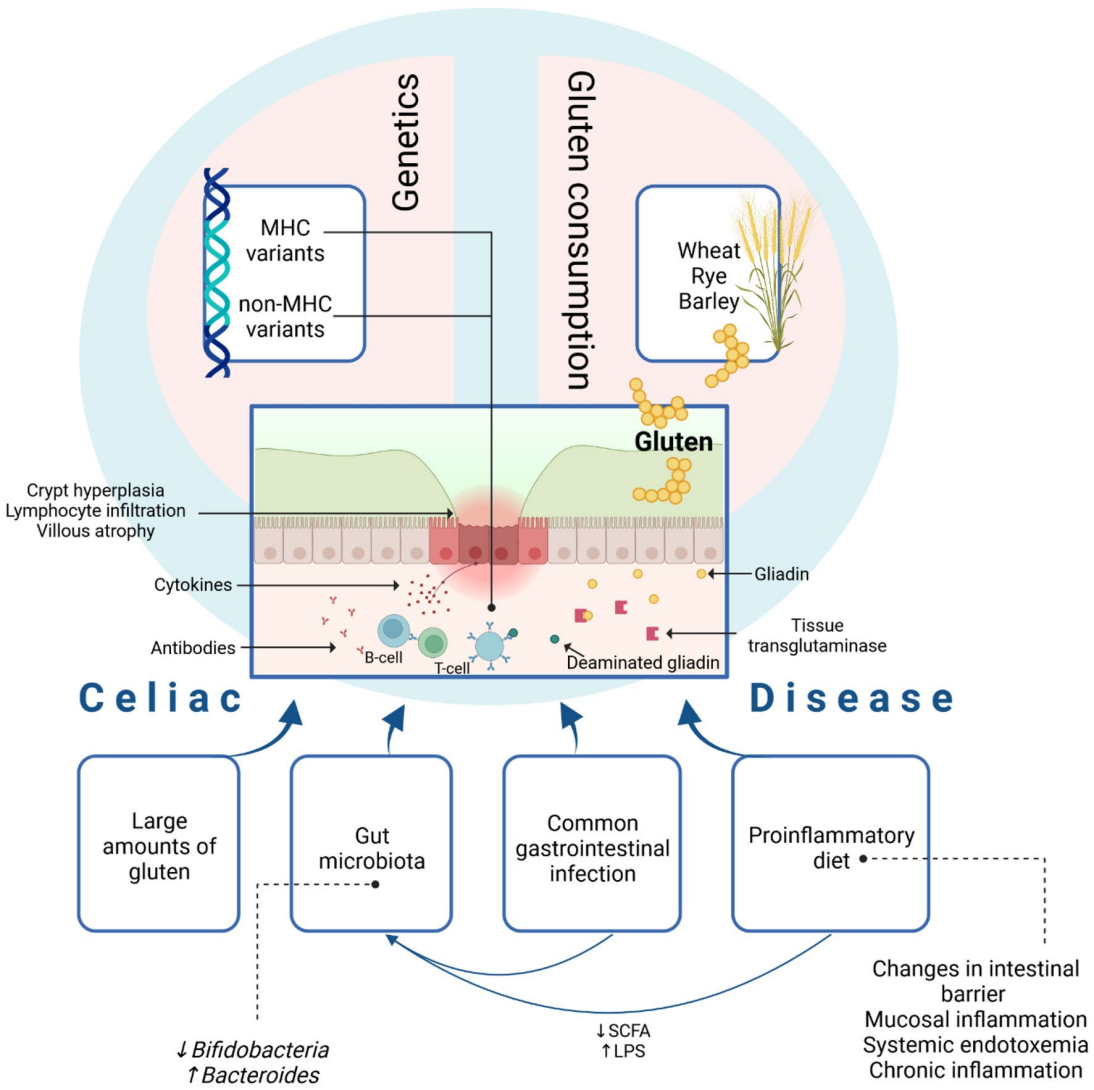
Pathogenesis of celiac disease. MHC—major histocompatibility complex; HLA—human leukocyte antigen; SCFA—short-chain fatty acids; LPS—lipopolysaccharide. Exposure to gluten may activate cell-mediated and humoral immune response in genetically predisposed individuals leading to the crypt hyperplasia, lymphocyte infiltration and villous atrophy in the small intestine. Tissue transglutaminase deaminates gliadin, resulting in greater proliferative response of gliadin-specific T-cells, leading to mucosal inflammation and B-cell activation

Celiac disease (CD) is a chronic small intestinal immune-mediated enteropathy precipitated by exposure to dietary gluten in genetically predisposed individuals [1]. In the submucosa of the small bowel, the enzyme called tissue transglutaminase (tTG) deamidates gluten peptides, leading to high-affinity binding to human leucocyte antigen (HLA) DQ2 and HLA DQ8 molecules. In patients with CD, this mechanism triggers an inflammatory reaction followed by tissue damage [2]. Factors predisposing to the development of CD are presented in Fig. 1.

The worldwide prevalence of CD is estimated to be 0.7–1.4% of the general population [3]. This prevalence can be different from continent to continent and country to country. In Europe, a higher prevalence has been reported in northern (1.6%) compared to eastern (0.98%), southern (0.69%), and western (0.60%) countries [4]. While the small bowel is the primary target of the immune response, CD is now known as a systemic disease as its manifestation embraces different organs, including the musculoskeletal apparat [5].

### Definition and Epidemiology of Osteoporosis in the Course of Celiac Disease

According to the American College of Physicians, osteoporosis is a skeletal disorder resulting in low bone mass and micro-architectural deterioration in bone tissue, which further leads to increased bone fragility and a higher risk of bone fractures. Celiac disease—both classic and asymptomatic—is one of the risk factors for development of secondary osteoporosis [6].

In 1994 World Health Organization (WHO) proposed general categories of osteoporosis for women based on measurements by dual-energy X-ray absorptiometry (DEXA) [7]. Osteoporosis is diagnosed when bone mineral density (BMD) is 2.5 SD or more below the average value for young healthy women and osteopenia when BMD is more than 1 SD below the young adult mean, but less than 2.5 SD below this value.

A modern view of osteoporosis includes in its definition the occurrence of previous fractures and other risk factors [8, 9]. Therefore risk assessment algorithms like FRAX [10] include in their calculation conditions of secondary osteoporosis including celiac disease. Although pathophysiological mechanisms underlying bone disorders in CD are not fully understood, possible mechanism include malabsorption of calcium, vitamin D, and other crucial for bone health nutrients as a result of villous atrophy and inflammation in small intestine. Also inflammation and production of proinflammatory cytokines drive bone tissue degradation. These mechanisms are fully discussed below.

The prevalence of osteoporosis seems to be 18.3% worldwide with the higher prevalence in Africa (39.5%) affecting both women and men—23.1% and 11.7%, respectively [9]. The consequence of osteoporosis are fractures with countless impact on healthcare spending. Osteoporosis affects up to 70% of CD patients at the diagnosis but generally is reported to be under 20% and osteopenia under 40%, although test results very often vary [6]. The limitation and probably the reason for these differences is the fact that in most studies these estimations do not take into account the presence of other risk factors like age or endocrine disorders. On a group of 563 premenopausal women and men, after excluding other factors predisposing to the development of osteoporosis, such as smoking, it was estimated that osteoporosis occurs in 14.4% and osteopenia in 39.6% of patients [6].

In the latest systematic literature review, Mosca et al. observed a high prevalence of low BMD in newly diagnosed patients between 20 and 35 years [11]. Moreover, peak bone mass is reached between age 20–30 years, so it can impact risk of later osteoporosis. A progressive bone loss starts at age 35 and reaches peak after 50 years of life. It affects mainly women [12].

CD-related osteoporosis can lead up to 20–25% of peripheral bone fractures in those patients, especially involving the wrist [13], and can variably involve the hip, lumbar spine, and distal radius [14]. Most bone fractures occur before the diagnosis of CD confirming that osteoporotic fractures can be a sign of an undiagnosed CD.

**Fig. 2:**
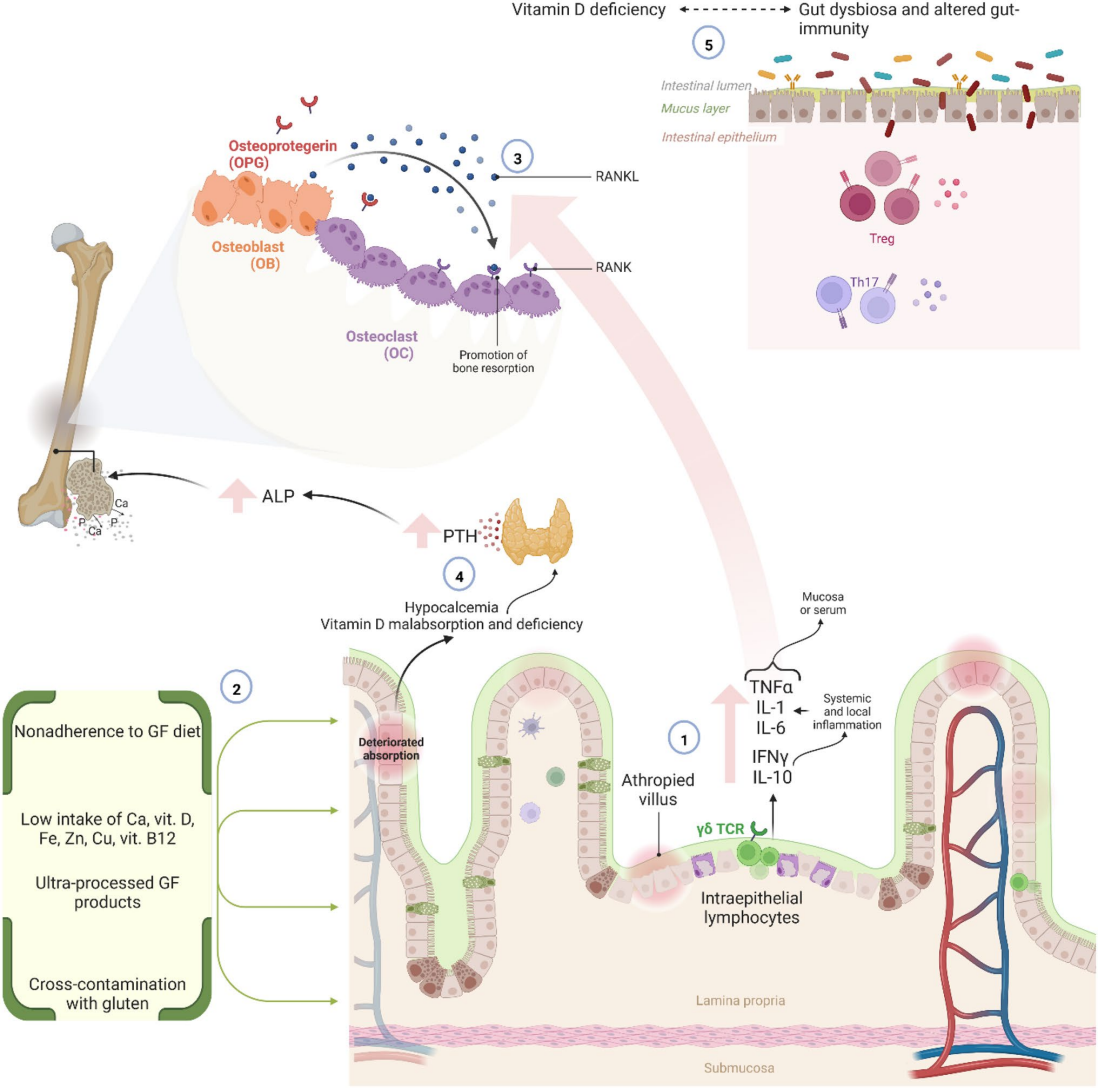
Pathogenesis of osteoporosis in the course of celiac disease. GF—gluten-free; Ca—calcium; vit.- vitamin; Fe—iron; Zn—zinc; Cu—copper; PTH—parathyroid hormone; ALP—alkaline phosphatase; γδ TCR—gamma-delta T-cell receptor; IL—interleukin; IFNγ—interferon gamma; TNFα—tumor necrosis factor α; RANKL—receptor activator for nuclear factor κ B Ligand; RANK—receptor activator of nuclear factor-κB; Treg—regulatory T cells; Th17—T helper 17 cell. 1. Increased number of intraepithelial lymphocytes with gamma-delta T-cell receptors leads to higher expression of several cytokines, e.g., INF-ϒ and IL-10, which results in local and systemic inflammation affecting bone mass in patients with celiac disease. Moreover, cytokines may stimulate osteoclastogenesis and recruitment of osteoclasts (affect RANKL/RANK/OPG pathway. 2. Non-adherence to GF diet—including cross-contamination with gluten—or including primarily ultra-processed, GF products can lead to nutrient deficiencies, affecting bone metabolism. Moreover, frequently observed low concentrations of serum vitamin D and calcium may increase serum concentrations of PTH (aspect discussed in part 4). 3. RANKL—which acts through a RANK receptor—is produced by mature osteoclasts and induces differentiation of osteoclasts, promoting bone resorption. On the other hand, osteoprotegerin—secreted by osteoblasts—has anti-osteoclastogenesis properties through inhibiting RANKL-RANK binding. 4. Low concentrations of vitamin D accompanied with impaired calcium absorption and low dietary intake, may lead to hyperparathyroidism, resulting in increased PTH levels and therefore to increased bone resorption. 5. Gut dysbiosis—through regulation of the immune system—seems to play an essential role in bone-mineral metabolism and osteoporosis. Moreover, gut dysbiosis may be associated with impaired absorption of vitamin D, increasing the risk of osteoporosis.

Importantly, BMD improves after introducing a strict GFD [15]. While CD patients are known to have a high prevalence of osteoporosis, the actual dimension of bone fracture risk is more controversial, especially after diagnosis [16–18]. A higher risk of fractures of any type seems to be more than plausible in CD patients both before and after the diagnosis, but difficult to estimate precisely [19]. Given those premises, the advances in diagnosis of CD may have mitigated the prevalence of fractures in CD patients in the XXI century [20]. Factors predisposing to the development of osteoporosis in patients with celiac disease are presented in Fig. 2.

It is worth to mention that studies on groups of women with CD show that their physical activity rates are at very low levels, presumably as a result of symptoms and complications of the disease what may also translate into a risk of decreased bone mineral density [21, 22].

### Genetic and Immunological Conditions of Osteoporosis in the Course of Celiac Disease

The correlation between bone disorders and celiac disease was noticed decades ago [23]. Osteoporosis deserves special attention as a non-celiac symptom of CD; in fact, abnormal bone metabolism and decreased BMD may be the only signs of “silent” CD [24]. Newly diagnosed patients or those inadequately treated for celiac disease often suffer from bone disorders, reduced bone mass, and BMD, consequently leading to increased bone fragility [24, 25]. Interestingly, studies have shown that osteoporosis has a worse course in those affected by CD [26] and presents as a extraintestinal manifestation of the disease. Scientific reports also indicate that osteoporosis is more common in people with celiac disease (3.4%) than in those without celiac disease (0.2%). In addition, severe cases of CD are accompanied by more severe osteoporosis [27], and people with celiac disease, who also suffer from reproductive disorders, are affected even more severely [26]. To date, it has been shown that about 75% of celiac patients also suffer from bone mass loss associated with osteopenia or osteoporosis [28–32]. Despite this strong association between low BMD values and CD, there are currently no guidelines on whether every patient with newly diagnosed CD should undergo DEXA scanning [33].

One of the theories of the development of osteoporosis in the course of celiac disease is due to a characteristic pathophysiological feature of celiac disease—an increased number of intraepithelial lymphocytes with gamma-delta T-cell receptors compared to healthy individuals [11]. These lymphocytes show significantly higher expression of interferon-gamma (INF-ϒ), which is the predominant cytokine in the damaged intestinal mucosa, and interleukin-10 (IL-10) [34]. Thus, it follows that both local and systemic inflammation plays a key role in bone mass loss in CD, characterized by an increase of pro-inflammatory cytokines in mucosa and serum, particularly tumor necrosis factor-alpha (TNFα), interleukin-1 (IL-1), and interleukin-6 (IL- 6), which has been confirmed [35, 36]. TNFα and IL-1 stimulate osteoclastogenesis and cause bone resorption and IL-6 recruits osteoclasts and promotes their differentiation [37]. Another study showed that increased production and release of TNFα and INF-ϒ is associated with increased bone mass loss. Higher levels of the cytokines TNFα and also IL-1, IL-6 stimulating osteoclasts were also observed in CD patients, with concomitant low levels of the cytokines interleukin- 18 (IL-18) and interleukin-12 (IL-12) [38, 39]. These events can directly affect bone density, independent of vitamin D levels and parathyroid hormone (PTH) levels.

The second theory assumes a lack of vitamin D and an impaired calcium absorption mechanism, leading to secondary hyperparathyroidism. Patients with celiac disease have lower vitamin D levels correlating with an increase in PTH levels [40] consequently leading to increased bone resorption (raises alkaline phosphatase levels), which explains the patient's symptoms.

In recent years, much attention has been paid to the receptor activator of NF-κB ligand(RANKL)/receptor activator of nuclear factor-κB (RANK)/osteoprotegerin (OPG) [RANKL/RANK/OPG] pathway, which is now considered as a major signaling system in bone metabolism. The expression level of the *OPG* gene is affected by many factors: cytokines (TNF-α, IL-1α, IL-18, TNF-β), bone morphogenetic proteins (BMP), 17β-estradiol, tensional forces; glucocorticosteroids, immunosuppressants, PTH, prostaglandin E2, or fibroblast growth factor (FGF). RANKL is produced by mature osteoblasts and their precursors, as well as by activated T lymphocytes [41]. Its expression is influenced by cytokines (IL-1, IL-6, IL-11, TNF-α), glucocorticosteroids, as well as PTH and 1,25(OH)vitamin D3, among others (8). It is a factor that activates the entire process of mature osteoclast formation: through differentiation, fusion, function, and survival of resorption cells bone (6). The genesis of such pathological bone changes in celiac disease originates in the mucosa of the small intestine, most severely affected by the disease. Inflammation of the mucosa from the duodenum to the distal part of the ileum lead to malabsorption and hepatic steatosis, which consequently translates into bone disorders. However, the mechanism is not entirely so straightforward. The consequences of bone disorders lie primarily in interactions between cytokines and factors affecting bone formation and resorption.

In patients with the asymptomatic form of celiac disease, factors correlated with chronic CD (i.e., growth factor deficiency, increased cytokine production, and autoimmune changes) may be the main factors leading to decreased BMD [37, 42]. A study by Moreno et al. suggests that low BMD in the total skeleton of CD patients is associated with an allelic variant of the *IL1B* gene. This implies that carrying IL1B-511 T is associated with lower bone mass in the peripheral skeleton of adult CD patients [43] and an increased prevalence of osteopenia/osteoporosis, which could explain one of the causes of bone weakness in CD [37].

### Nutritional Determinants of Osteoporosis in the Course of Celiac Disease

Loss of villous cells in the proximal intestine leads to deterioration of intestinal absorption efficiency. As a result, patients who do not adhere to a GFD have a high risk of nutrient and vitamin deficiencies, including calcium and vitamin D essential for proper bone metabolism [44]. Hypocalcemia and vitamin D malabsorption lead to compensatory increases in serum levels of PTH, which is observed in newly diagnosed individuals with CD. After the implementation of a strict GFD which results in the reconstruction of intestinal villi, PTH serum levels normalize [45].

In addition, in patients with the classic form of celiac disease, manifested by intestinal symptoms, observed is the reduction of intestinal calcium absorption due to its binding to unabsorbed fatty acids, and as a result, increased fecal calcium losses and decreased urinary excretion of calcium [44, 46].

Also, deficiencies in other minerals and vitamins can adversely affect bone metabolism. Common nutritional deficiencies in the newly diagnosed and untreated CD are iron, magnesium, B12, calcium, vitamin D, zinc, and copper [47]. For example, zinc deficits correlate with low levels of insulin-like growth factors, that are responsible for alterations in bone metabolism [48, 49]. After the introduction of a gluten-free diet, the values return to normal in most patients, however, approximately 30% of patients with CD nutritional deficiencies can persist [47].

Magnesium, as intracellular cation, is necessary for calcium metabolism. Its 50–60% of the total body content is accumulated in the bone where its ions bind at the surface of the hydroxyapatite crystals, improve the solubility of phosphorous and calcium hydroxyapatite. Magnesium induces osteoblast proliferation and is also necessary for the activation of vitamin D because most of the enzymes involved in vitamin D metabolism require magnesium [50].

Nevertheless, introducing a gluten-free diet has a positive effect on BMD in patients with CD [28, 30, 32, 51–55]—although not all of them manage to restore BMD adequate for healthy people. Zanchetta et al. conducted an interesting study in this area. Researchers assessed BMI, bone microarchitectural parameters including BMD, and biochemical measurements—IgA tTG, IgA DGP, IgG DGP, hemoglobin, calcium, C-telopeptide, PTH, 25OHD in patients at diagnosis and after 1 year on GFD and supplementation of vitamin D and calcium. The study showed that after 1 year of treatment, the bone microarchitecture improved, yet there were still significant differences with a control group of women of similar age and BMI [56].

Thus, on the one hand, strict exclusion of gluten from the diet is the only possible form of CD treatment, and strict adherence to it allows for the reconstruction of damaged intestinal villi and, as a result, improvement of intestinal absorption [49]. On the other hand, a gluten-free diet is very often a diet deficient in minerals and vitamins that are essential for bone health. Additionally, despite strict adherence to diet, patients are at risk of cross-contamination with gluten [49].

In addition, patients with CD very often focus on excluding gluten from their diet, forgetting the importance of a proper balance of the menu in terms of the content of minerals and vitamins. Research indicates that the diet of patients with CD is low in cereals, fruits, and vegetables and excessive in meat and derivatives. Furthermore, it is very often based on processed, gluten-free, low-nutritional products [57–59]. Gluten-free products are characterized by a higher content of saturated fats, simple sugars and a lower content of vitamins and minerals than their traditional counterparts [49]. Processed foods and an improperly balanced, high-fat diet also promote the development of low-grade chronic inflammation [60]. As we know that BMD of patients is largely determined by the ongoing inflammation, it is also worth paying attention to the quality of the patient's diet in terms of its inflammatory nature [61].

It should also be noted that many celiac patients limit or even exclude the consumption of dairy products. Most of the CD patients with villi atrophy experience secondary lactose malabsorption due to decreased lactase activity on the enterocyte brush border. In a study by Ojetti et al. 24% of patients with lactose intolerance determined by HBT were diagnosed as CD and in many, lactase deficiency was the only manifestation of CD [62]. However, it should be noted that after introducing a gluten-free diet in CD patients, intestinal villi begin to rebuild, and after 1–2 months in most patients, lactose begins to be well tolerated again [47].

The elimination of milk and milk products from the diet, which are one of the richest sources of calcium, is considered one of the possible causes of the frequent occurrence of reduced BMD in patients with CD [63]. Unfortunately, many CD patients, as well as the general population, avoid dairy in their diet – in the study by Zingone et al. 22.2% of patients with CD and 19.9% of healthy patients constituting the control group claimed not drinking fluid milk regularly [64]. Therefore, the nutritional education of patients is of key importance, including not only guidelines for the elimination of gluten from the diet, but also the correct composition and balancing of the diet depending on the coexisting symptoms.

### Gut Microbiota and Osteoporosis in the Course of celiac disease

The gut microbiota has a pivotal role in determining immune self-tolerance and, over the years, it has become clear its importance in the complex pathogenesis of autoimmune diseases [65]. Through microbial translocation and molecular mimicry, the microbiota regulates both innate and adaptive immune homeostasis of the local and systemic immune system [66]. Immune dysregulation due to altered gut microbiota and infections have been advocated as possible explanation for the increasing prevalence of autoimmune diseases during XX and XXI centuries but other interpretations are possible [67].

Recent evidence suggest an important role of the gut microbiota in bone-mineral metabolism and osteoporosis [68, 69]. Once more, this influence of gut microbiota seems to be played through its regulation of the immune system [70]. The altered immune-inflammatory axis due to the impaired gut microbiota could partially explain the higher prevalence of osteoporosis in autoimmune diseases regardless of corticosteroids and immunosuppressants. The connection between autoimmunity, gut microbiota, and BMD it is only partially justified by the activity of T lymphocytes helper Th17 and regulatory T (Treg) [71]. Vitamin D would play a bivalent role in the connection between the gut microbiome and bone metabolism [70]: vitamin D has a role in regulating the microbiome and the immune-inflammatory axis (Treg) but, at the same time, an altered microbiome and inflammatory diseases (i.e., IBDs and CD) can lead to a reduced vitamin D absorption. For all these reasons, gut microbiota is thought to be a possible future target of osteoporosis treatment [69].

In particular, CD and IBDs share many common patterns of the gut microbiome [72]. The taxa included in those gut microbiomes were found to be associated with low BMD, strengthening the association between autoimmune diseases, and osteoporosis. The gut microbiome is thought to be crucial in the normalization of BMD after the initiation of a GFD [15]. More studies are needed to better understand the precise role of the microbiome in autoimmunity and osteoporosis and its implications on therapy.

### Environmental Determinants of Osteoporosis in the Course of Celiac Disease

Physical activity (PA) has a positive effect on bone metabolism and has a preventive effect on the development of osteoporosis [73]. Peak bone mass is achieved by late adolescence, therefore, promoting physical activity is particularly important among children and adolescents [74]. The National Osteoporosis Foundation and International Osteoporosis Foundation recommend weight-bearing exercises in the prevention of osteoporosis—especially high-intensity PA such as jumping, hopping, but, mostly in adults, also lower-intensity PA as walking and weight training is recommended [73].

An interesting study was conducted by Nestares et al. to evaluate of influence of Mediterranean Diet adherence and PA on body composition and bone health in young patients with CD. They observed in a group of 59 children with CD and 40 healthy controls that patients with CD have lower body weight, lean mass, and bone mass than non-celiac children, regardless of the length of time on a GFD [75]. Furthermore, they observed that lean mass is independently associated with BMD in these patients, explaining 11% of its variability [75]. Studies suggest that BMI can be considered as a predictor of bone density in children with CD since lower BMI is associated with lower BMD [76, 77].

Attention should be also paid to toxins and environmental pollution, since studies show that environmental substances may be related to osteoporosis [78]. In a population-based, cross-sectional study analysis was made of data collected from the National Health and Nutrition Examination Survey. The authors observed that 115 persons following a GFD had significantly higher urine levels of total arsenic and blood levels of mercury, lead, and cadmium than 11 239 persons not avoiding gluten. These results suggest that a gluten-free diet that may contribute to the accumulation of potentially toxic metals [79, 80]. In the meta-analysis, the authors found that exposure to cadmium and lead, but not mercury, is associated with an increased risk of osteopenia or osteoporosis in adults [81].

## Therapeutic Strategies

### Lifestyle and Dietary Modifications

Osteoporosis treatment for patients with CD includes non-pharmacological and pharmacological interventions. A strict gluten-free diet reduces systemic inflammation, heals intestinal mucosa, allows recovery of nutrient absorption and, as a result, improvement or normalization of BMD [82, 83]. However, it should be emphasized that, apart from the elimination of gluten from the diet, it is crucial to properly balance patient’s diet and rely on products with high nutritional density. It is worth educating patients and explaining the role of an appropriate diet and lifestyle in the prevention of osteoporosis—Quattrini et al. conducted a prospective observational study in a population of 200 women aged 30–80 years and observed that patients who follow MD, consume more dietary calcium. Furthermore, the authors underline that short nutritional conversation improves lifestyle habits that could have a preventive effect on osteoporosis development [84]. Numerous studies confirm the bone benefits of the MD in the prevention of osteoporosis in the general population. Data from 49 cross-sectional, case–control, longitudinal, and clinical trial studies suggest that a plant-based diet, rich in whole grains, poultry, fish, nuts, legume, and low-fat dairy products have a positive impact on bone health. It seems to be a positive outcome of the high nutritional density of these products—for example, fruits and vegetables are rich in potassium, magnesium, vitamin C, vitamin K, folate, carotenoids, fish and seafood abound in anti-inflammatory omega-3 fatty acids, and dairy products are the best dietary source of calcium and magnesium in the diet. On the contrary, the western-style diet—characterized as a high-caloric diet, rich in refined grains and sugar, e.g., soft drinks, sweets, and desserts, sodium, phosphorus, saturated fats, animal protein, e.g., meats and derivatives, and low in fiber, vitamins, and trace elements—have been proven to be a deleterious for bones [85].

It is crucial to consume adequate amounts of calcium—dairy products, e.g., milk, yogurt and cheese, fish—especially sardines with bones, pulses, nuts, and seeds [44]. If dietary calcium requirements cannot be met, adequate supplementation with calcium should be provided [82]. Furthermore, care should be taken to maintain the appropriate concentration of vitamin D. Since only 10–20% of vitamin D is obtained from a foods—such as oily fish, mushrooms, or fortified dairy products—the role of moderate sun exposure is emphasized, because the total of 80–90% of vitamin D is obtained from cutaneous synthesis [50]. Adequate vitamin D supplementation should be provided [86]. Special attention should also be paid to potassium and magnesium, vitamin K, C, omega-3 fatty acids, folate, vitamin B12, and zinc, as well as an adequate supply of wholesome protein [50].

A very interesting study in this area on a group of 57 children with CD and 40 healthy children was conducted by Nestares et al. The authors observed that higher MD adherence and more time performing vigorous physical activity were associated with higher Z-score and lean mass among CD children [75]. Therefore, it is also advisable to ensure an appropriate level of physical activity, which promotes better inflammatory and redox profiles in the general adult population. Patients should be encouraged to participate in weight-bearing exercises, but also limit alcohol intake, and avoid cigarette smoking [82].

It is also important to identify early risk factors for the development of osteoporosis, such as estrogen deficiency and hypoghonadism.

### Clinical Treatment of Osteoporosis

There is no general agreement on the correct timing of bone densitometry in celiac patients. According to the European Society for the Study of Coeliac Disease (ESsCD), bone densitometry should be performed in every patient at the time of diagnosis and should be repeated after 2–3 years if abnormal—or in patients who have evidence of ongoing villus atrophy or do not adhere strict gluten free diet—or 5 years if normal [87]. Furthermore, it is recommended to measure calcium, alkaline phosphatase and vitamin D levels at diagnosis and replace as necessary [87]. Other guidelines suggest performing bone densitometry only in patients with a high risk of osteoporosis or those older than 55 years [83, 88, 89].

Pharmacological treatment of osteoporosis in patients with celiac disease includes taking bisphosphonates, denosumab, teriparatide, romosozumab. There are no longitudinal studies dealing with effect of particular form of osteoporosis treatment in patients with CD and there are no studies on which to base the choice in the individual subgroups of patients.

Bisphosphonates usually are first line treatment in treating ostoporosis. They act by inhibiting farnesyl pyrophosphate synthase which is an osteoclast enzyme. Bisphosphonates promote apoptosis of osteoclasts and, as a result, stop bone resorption and increase BMD. According to ESsCD, it is recommended to start calcium and vitamin D supplementation before treatment with bisphosphonates to decrease risk of tetany in patents with osteomalacia [87]. In celiac disease patients, parenteral treatment options such denosumab or intravenous bisphosphonates, e.g., zoledronic acid are preferred above oral medication due to poor absorption of oral bisphosphonates. Other options include follow-up bone markers such as CTX—bone resorption—and P1NP—bone formation—assessment after 3–6 months of therapy to assess efficacy of absorption [90].

Denosumab is a monoclonal antibody to RANKL and romosozumab is a monoclonal antibody to sclerostin which blocks canonical Wnt signaling bone formation pathway. Obstacles associated with romosozumab use include the high cost and the need for monthly appointments to receive a dose of the drug [91]. Lastly, teriparatide is the first drug in category of osteoporosis called anabolic therapy [92]. It is recombinant parathyroid hormone, reserved for severe osteoporosis with high risk of fractures, that increase bone formation [90].

## Summary and Conclusion

Celiac disease is characterized by inflammation of the small intestine and villous atrophy [1]. Small intestinal villous atrophy leads to impaired nutrient absorption, acute weight loss, and diarrhea, as well as fractures. A 2015 meta-analysis demonstrated that celiac patients have a 30% higher risk of any fracture and a 69% higher risk of femoral neck fracture [19]. Another meta-analysis from 2017 showed that patients with CD had an increased risk of osteoporosis [93]. Similar results were also obtained in large screening studies [94]. The conclusion of such studies would seem fairly straightforward; however, these studies did not examine the risk of CD in patients who were first diagnosed with osteoporosis, which is important for clinicians because of the decision to screen a patient with osteoporosis for celiac disease.

The paper by Laszkowska and colleagues, in which they analyzed articles published in PubMed, Medline, or EMBASE up to May 2017 to estimate the prevalence of celiac disease in patients with osteoporosis, identified eight studies, among which 3188 osteoporotic subjects were identified, 59 of whom (1.9%) had CD. Also weighted pooled analysis showed biopsy-confirmed celiac disease in 1.6% of people with osteoporosis. Importantly, with the inclusion of studies based on positive celiac serology—the absolute percentage increased from 1.9 to 2.6%, but the total weighted prevalence remained at 1.6% [95].

Various studies have shown that treatment with a gluten-free diet improves BMD in celiac patients suggesting that treating celiac disease would likely reduce the excess risk of fractures, but it is important to remember that the mechanism of osteoporosis in celiac patients is likely multifactorial. Individuals with celiac disease at the time of diagnosis often have low bone density, even in the absence of celiac disease symptoms, which may be due to impaired intestinal absorption efficiency, leading to hypocalcemia and vitamin D malabsorption [40]. This in turn results in secondary hyperparathyroidism, as well as chronic inflammation causing increased production of pro-inflammatory cytokines in the mucosa and serum, particularly TNFα, IL-1, and IL-6 [11] disrupting bone growth and autoimmune factors.

In addition, the quality of patients' diets is worth considering since research show that patients with CD consume low amounts of cereals, fruits, vegetables and excess of meat and derivatives [57–59]. Gluten-free products are often highly processed products characterized by a higher content of saturated fats, simple sugars and a lower content of vitamins and minerals than their traditional counterparts [49].

Recent evidence also suggests an important role for the gut microbiota in bone metabolism, but more research is needed to better understand the exact role of the microbiome in osteoporosis in CD [68, 69]. It appears that physical activity and environmental pollution may also influence the development of bone disorders in CD [75, 81].

Answering the question in the title, which factor—genetic, immunological, dietary, gut microbiota, or environmental—plays the first violin in the development of osteoporosis in the course of celiac disease, we emphasize that there is no unequivocal answer to the question. All the presented determinants are important elements that affect the development of bone disorders in patients with celiac disease, and abnormalities in one can disrupt the entire complex mechanism of action.
